# Breast cancer examination uptake and its associated factors among women in East African countries: A systematic review and meta-analysis

**DOI:** 10.1371/journal.pgph.0006727

**Published:** 2026-11-05

**Authors:** Getaneh Awoke Yismaw, Mekuriaw Nibret Aweke, Meron Asmamaw Alemayehu, Nebiyu Mekonnen Derseh, Tigist Kifle Tsegaw, Tilahun Yemanu Birhan, Banchlay Addis, Eyob Akalewold Alemu, Fetlework Gubena Arage, Gebrie Getu Alemu, Habtamu Abebe Getahun, Wagaye Atalay Taye, Habtamu Wagnew Abuhay

**Affiliations:** 1 Department of Epidemiology and Biostatistics, Institute of Public Health, College of Medicine and Health Sciences, University of Gondar, Gondar, Ethiopia; 2 Department of Human Nutrition, Institute of Public Health, College of Medicine and Health Sciences, University of Gondar, Gondar, Ethiopia; 3 Department of Health Systems and Policy, Institute of Public Health, College of Medicine and Health Sciences, University of Gondar, Gondar, Ethiopia; 4 Department of Clinical Pharmacy, School of Pharmacy, College of Medicine and Health Sciences, University of Gondar, Gondar, Ethiopia; University of Embu, KENYA

## Abstract

Breast cancer is a global public health issue and the leading cause of cancer-related morbidity and mortality among women. Its burden is rising rapidly in low- and middle-income countries. In resource-limited settings, clinical breast examination (CBE) offers a feasible and cost-effective screening method for early detection. Despite its importance, the regional evidence on CBE uptake and its determinants in East Africa remains limited. Therefore, this study aimed to estimate the pooled uptake of CBE and its associated factors among women in the region. This study adhered to the PRISMA 2020 guidelines and included all observational studies published between January 1, 2015, and April 30, 2025. Databases such as PubMed, Scopus, Research4Life, and Google Scholar were searched. Publication bias was assessed using the funnel plot and Egger’s regression analysis. Furthermore, subgroup and sensitivity analyses were performed. A total of 51, 100 participants from fifteen studies were included in this analysis. The pooled uptake of CBE in East African countries was 15% (95% CI: 12%, 18%). Women aged 35–49 years (AOR = 2.84; 95% CI: 1.57, 5.13), higher educational attainment (AOR = 2.31; 95% CI: 1.48, 3.60), and having awareness of breast cancer screening methods (AOR = 2.05; 95% CI: 1.23, 3.28) were more likely to uptake CBE services. However, rural residence was associated with lower odds (AOR = 0.82; 95% CI: 0.68–0.99) of CBE. Clinical breast examination uptake was low women in East Africa is low. Higher CBE uptake was associated with being aged 35–49, higher education, and awareness of screening methods; while rural residence was associated with lower uptake. National policy makers and stakeholders should consider prioritize raising awareness and embedding screening into primary healthcare, particularly for rural, younger, and lower educational level women to enhance CBE uptake.

## Introduction

Breast cancer is public health concern worldwide and the main cause of cancer-related morbidity and mortality in women [1]. Globally in 2024, 2.4 million women were diagnosed with breast cancer, resulting in 694,000 deaths, while 8.2 million women were living with breast cancer [2]. There are substantial variations in breast cancer incidence and mortality worldwide [3].

Clinical breast examination, defined as physical examination of the breasts performed by a healthcare provider to detect lumps or other abnormalities [4]. Breast cancer is becoming increasingly prevalent in low- and middle-income nations [5], notably in Sub-Saharan Africa [6]. In Africa, an estimated 38 per 100,000 women were diagnosed with the disease in 2022, with 19 per 100,000 dying from it [7]. Approximately 70% of diagnosed women in African countries have advanced-stage illness, which significantly decreases their chances of survival [8], and early detection of breast cancer is crucial for better outcomes [9].

The World Health Organization (WHO) emphasized the necessity of promoting cancer early detection strategies, particularly in resource-limited settings [10]. Clinical breast examination (CBE) is a practical and cost-effective screening method that entails a thorough physical examination of the breasts by a qualified healthcare professional [11]. CBE is crucial for early detection of breast abnormalities and is especially valuable in low-resource settings where mammography is not readily available [5,12].

An overview of systematic reviews evidenced that CBE is crucial for low- and middle-income countries where mammography program are impractical; whereas its uptake is low in high-income countries due to widespread access to mammography [12].

Despite its proven utility, the uptake of CBE remains low in many East African countries, ranging from 5.2% in a study from Tanzania [13] to 36.7% in Kenya [14]. The underutilization of CBE services contributes to delayed diagnosis, which increases the likelihood of disease progression to advanced and metastatic stages, resulting in poorer treatment outcomes and higher healthcare costs [15,16]. Although breast cancer is an increasingly significant public health issue [7], there is a paucity of comprehensive data on screening uptake and its associated determinants in East Africa.

Estimating the pooled CBE uptake and its determinants provides key evidence to track progress towards sustainable development goad (SDG) 3.4 in East Africa, which aims to reduce premature mortality from non-communicable diseases by one-third by 2030 through prevention and treatment [13,17].

Existing findings from primary cross-sectional studies on this topic show substantial inconsistency in prevalence rates and effect sizes. Because individual studies are frequently constrained by small, localized samples, a critical gap remains in the literature. The primary challenge is not a scarcity of raw data, but the lack of synthesized, critically appraised, and pooled evidence. A systematic review and meta-analysis is needed to resolve these inconsistencies, enhance statistical power, and establish a definitive, regionally representative estimate to inform clinical practice and public health policy.

Therefore, this study aimed to estimate the regional pooled uptake of CBE, identify associated factors across East African countries. The findings are expected to support targeted public health interventions to improve screening uptake, support policy formulation, and ultimately contribute to reducing breast cancer-related morbidity and mortality across the region.

## Methods and materials

### Study protocol and registration

The protocol for this systematic review and meta-analysis was registered in the International Prospective Registry of Systematic Reviews (PROSPERO) with registration number CRD420251003764. Study selection, data extraction, and reporting followed PRISMA 2020 guidelines [18].

### Search strategy and information sources

Databases such as PubMed, Scopus, Research4life, and Citation Manager (Google Scholar) were thoroughly searched for potential studies. We also searched Google for gray literature and reference lists of previously identified studies. The basic search terms and phrases were clinical breast examination, breast cancer screening, associated factors, and East African countries.

The MeSH terms, synonyms, search phrases, and Boolean operators were then identified and searched as follows: (((“clinical breast examination”[All Fields]) OR (“clinical breast cancer screening”[All Fields]) OR “breast cancer screening” [All Fields] OR “Early Detection of Cancer” [MeSH Terms] OR “early detection of breast cancer”[All Fields] OR “breast neoplasms”[MeSH Terms]) AND ((“Reproductive age women” [All Fields] OR “female”[All Fields]) OR (“women”[MeSH Terms] OR “women”[All Fields])) AND ((Burundi OR Comoros OR Djibouti OR Eritrea OR Ethiopia OR Kenya OR Madagascar OR Malawi OR Mauritius OR Mozambique OR Rwanda OR Seychelles OR Somalia OR “South Sudan” OR Tanzania OR Uganda OR Zambia OR Zimbabwe))). A search was undertaken for all papers published between January 1, 2015, and April 30, 2025 (S1 Table).

### Study design, sampling method and study population

We have explicitly clarified the study design as a systematic review and meta-analysis conducted in accordance with the Preferred Reporting Items for Systematic Reviews and Meta-Analyses (PRISMA 2020) guidelines. All observational primary studies involving women with age 15–49 years old were retrieved using all electronic databases and websites, as described in detail in the supplementary file (S1 Table). Following comprehensive screening, all included studies were found to be cross-sectional studies. We have refined the description of the study population using the CoCoPop (Condition, Context, and Population) framework. All studies meeting the eligibility criteria were included, without restriction on sampling methods. The included primary studies employed a variety of techniques, including simple random, systematic, stratified multistage, multi-stage random, cluster, and convenience sampling.

### Inclusion and exclusion criteria

This review included observational studies that reported the uptake, utilization, and prevalence of clinical breast examination, and/or studies that examined the determinants of CBE among women in East African countries. Studies were considered eligible if they were conducted in any of the following East African nations: Burundi, Comoros, Djibouti, Eritrea, Ethiopia, Kenya, Madagascar, Malawi, Mauritius, Mozambique, Rwanda, Seychelles, Somalia, South Sudan, Tanzania, Uganda, Zambia, and Zimbabwe. Studies published between January 1, 2015, and April 30, 2025, were included to post-2015 health system performance, modern policies, and SDG 3.4 benchmarks [19]. No language restrictions were applied in our search strategy.

However, review studies, case reports, editorial letters, articles without full text, and qualitative research were excluded from the review. We applied the CoCoPop framework (Condition, Context, Population) to structure the eligibility criteria (S2 Table)

### Outcome variables

The main outcome variable of this study was the uptake of clinical breast examination**,** defined as the proportion of women who had ever undergone physical examination of the breasts performed by a healthcare provider to detect lumps or other abnormalities [4]. The secondary outcome of the study was the identification of factors associated with CBE uptake**.** we utilized odds ratios (ORs) to assess the association between the uptake of CBE and a range of covariates, including age, residence, marital status, occupation, educational status, and women’s awareness of breast cancer.

### Study selection process and quality assessment

All articles retrieved from the databases were imported into EndNote version 21, and duplicate records were removed. Based on the predefined inclusion criteria, four authors (MNA, MAA, NMD, and TKT) independently screened the titles and abstracts of the identified studies. The screened results were then compiled, and any disagreements between reviewers were resolved through discussion with a fifth author (GAY).

The authors (TYB, BA, WAT, and HWA) subsequently evaluated the full texts of potentially eligible studies via the Joanna Briggs Institute (JBI) quality appraisal checklist [20]. Studies that scored 50% or higher on the quality assessment checklist criteria were considered to be of high quality and were included in this study (S3 Table). Data extraction was performed using the Joanna Briggs Institute (JBI) data extraction format. Finally, the Grading of Recommendations Assessment, Development and Evaluation (GRADE) approach was utilized, evaluating five core domains: risk of bias, inconsistency, indirectness, imprecision, and publication bias [21].

### Data extraction

Data were extracted independently by five authors (HWA, EAA, FGA, GGA, and HAG) using a structured Microsoft Excel spreadsheet. The extraction process followed the Joanna Briggs Institute (JBI) data extraction format [22], which included the following key elements: first author’s name, year of publication, country where the study was conducted, study design, sampling technique, response rate, total sample size, number of women who underwent CBE, and covariates related to the uptake of CBE were also extracted.

### Statistical analysis

All meta-analyses and statistical tests were conducted using STATA version 17. A random-effects using DerSimonian–Laird (DL) model to estimate the pooled uptake of CBE and to analyze factors associated with the uptake in East African countries. We used DL model because proportion of included studies were non-extreme (no variance instability near 0 or 1).

Publication bias was assessed using both subjective and objective methods [23]. A funnel plot was used for visual inspection, and Egger’s regression test was employed to statistically detect publication bias. A p-value < 0.05 was considered the presence of publication bias [24].

Heterogeneity among studies was assessed using the I^2^ statistic, which measures the percentage of total variation across studies. I^2^ values of 25%, 50%, and 75% were indicated as low, moderate, and high heterogeneity, respectively [25]. Following that, to identify potential sources of heterogeneity, subgroup analyses were performed based on study country, year of publication, sample size, study settings, residency and study quality. Finally, sensitivity analyses were conducted to assess the influence of individual studies on the overall pooled estimates and to identify any outliers that may contribute to heterogeneity.

### Ethical approval and consent

Ethical approval and consent to participate were not applicable for this study, as it was a systematic review and meta-analysis based on primary studies published on CBE in East African countries.

## Results

### Search results and included studies

A total of 1,953 articles were initially identified through a comprehensive search of multiple electronic databases. After removing 906 duplicate records, 1,047 articles remained for title and abstract screening. Of these, 1,026 were excluded based on their titles and abstracts, leaving 21 articles for full-text review. Following a thorough assessment, 15 studies met the predefined inclusion criteria and were included in the final systematic review and meta-analysis **(**Fig 1**)**.

**Fig 1:**
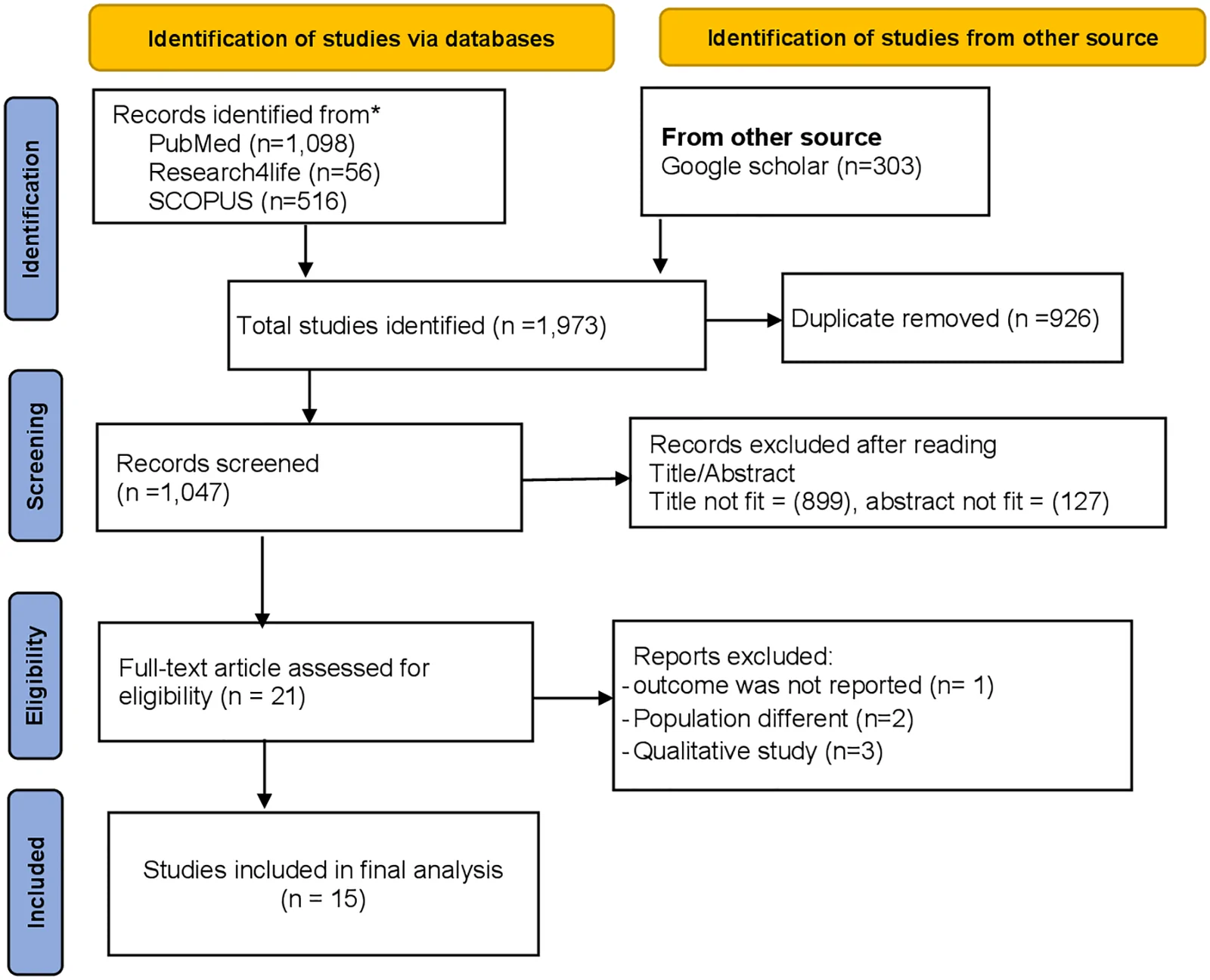
PRISMA 2020 flow diagram for systematic reviews and meta-analyses for clinical breast examination uptake among women in East African countries, 2025.

### Characteristics of the included studies

A total of 15 studies comprising 51,100 participants were included in this review and used to estimate the pooled uptake of CBE among women in East Africa. All of the studies employed a cross-sectional study design. There was considerable variation in sample size, ranging from 70 participants in a study conducted in Ethiopia [26] to 16,649 participants in a study conducted in Kenya [27].

Geographically, the studies were conducted across several East African countries: five studies were from Ethiopia [26,28-31], four from Kenya [14,27,32,33], and two from Rwanda [34,35], Tanzania [13,36], and Uganda [37,38]. The quality of the included studies was assessed using the Joanna Briggs Institute (JBI) critical appraisal checklist. Above 80% of the included studies were rated as high quality, suggesting a low risk of bias and enhancing the credibility of the synthesized findings (**Table 1)**. This study included 15 observational cross-sectional studies comprising 35,911 participants to estimate the pooled uptake of clinical breast examination (CBE) and identify its associated factors across East African countries. Following the Grading of Recommendations Assessment, Development and Evaluation (GRADE) framework, evidence derived exclusively from observational (cross-sectional) studies begins with a low-certainty rating and is evaluated across five core domains: risk of bias, inconsistency, indirectness, imprecision, and publication bias, which showed low certainty of evidence. We have updated in the supplementary file table (S4 Table).

**Table 1:** Characteristics of the included studies, JBI risk of bias assessment and prevalence of breast cancer screening uptake in East African countries, 2025.

Id	Author	Publication year	Country	Study setting	Place of residence	Sampling Strategy	Total Participant	Uptake of CBE (%)	Risk of bias
1	Abeje et al. [28]	2019	Ethiopia	Facility	Mixed	Simple random sampling	633	133 (21.0)	Low risk
2	Agide et al. [26]	2019	Ethiopia	Community	Mixed	Systematic random sampling	70	17 (24.3)	Moderate risk
3	Assefa et al. [29]	2021	Ethiopia	Community	Urban	Multistage sampling	634	37 (6.1)	Low risk
4	Dibisa et al. [30]	2019	Ethiopia	Community	Mixed	Systematic random sampling	422	24 (5.6)	Low risk
5	Ketema et al. [31]	2023	Ethiopia	Facility	Urban	Systematic random sampling	420	55 (13.0)	Low risk
6	Abuhay et al. [27]	2025	Kenya	Community	Mixed	Stratified Multistage sampling	16,649	2316 (13.9)	Low risk
7	Antabe et al. [32]	2020	Kenya	Community	Mixed	Cluster sampling	14,741	1768 (12)	Low risk
8	Maureen et al. [33]	2022	Kenya	Community	Urban	Multistage sampling	284	20 (7.1)	Low risk
9	Odhiambo et al. [14]	2022	Kenya	Community	Mixed	Systematic random sampling	319	116 (36.7)	Low risk
10	Igiraneza et al. [35]	2021	Rwanda	Facility	Mixed	Convenience sampling	246	36 (14.6)	Moderate risk
11	Chebet et al. [34]	2019	Rwanda	Facility	Urban	Consecutive sampling	384	98 (25.5)	Low risk
12	Joho et al. [36]	2024	Tanzania	Community	Urban	Systematic random sampling	354	52 (14.7)	Low risk
13	Tibenderana et al. [13]	2024	Tanzania	Community	Mixed	Stratified Multistage sampling	15,189	790 (5.2)	Low risk
14	Atuhairwe et al. [38]	2018	Uganda	Community	Mixed	Cluster sampling	414	150 (36.2)	Low risk
15	Sharp et al. [37]	2019	Uganda	Community	Mixed	Convenience sampling	341	53 (15.5)	Moderate risk

### Pooled uptake of clinical breast examination in East African countries

Based on a random-effects meta-analysis, the overall pooled uptake of clinical breast cancer examination among women in East Africa was 15% (95% CI: 12%, 18%). Heterogeneity across studies was substantial (I^2^ = 98.89%, *p-value* < 0.001). Reported uptake ranged from 5% in Tanzania to 36% in Uganda [13,37,38] (Fig 2**)**.

**Fig 2:**
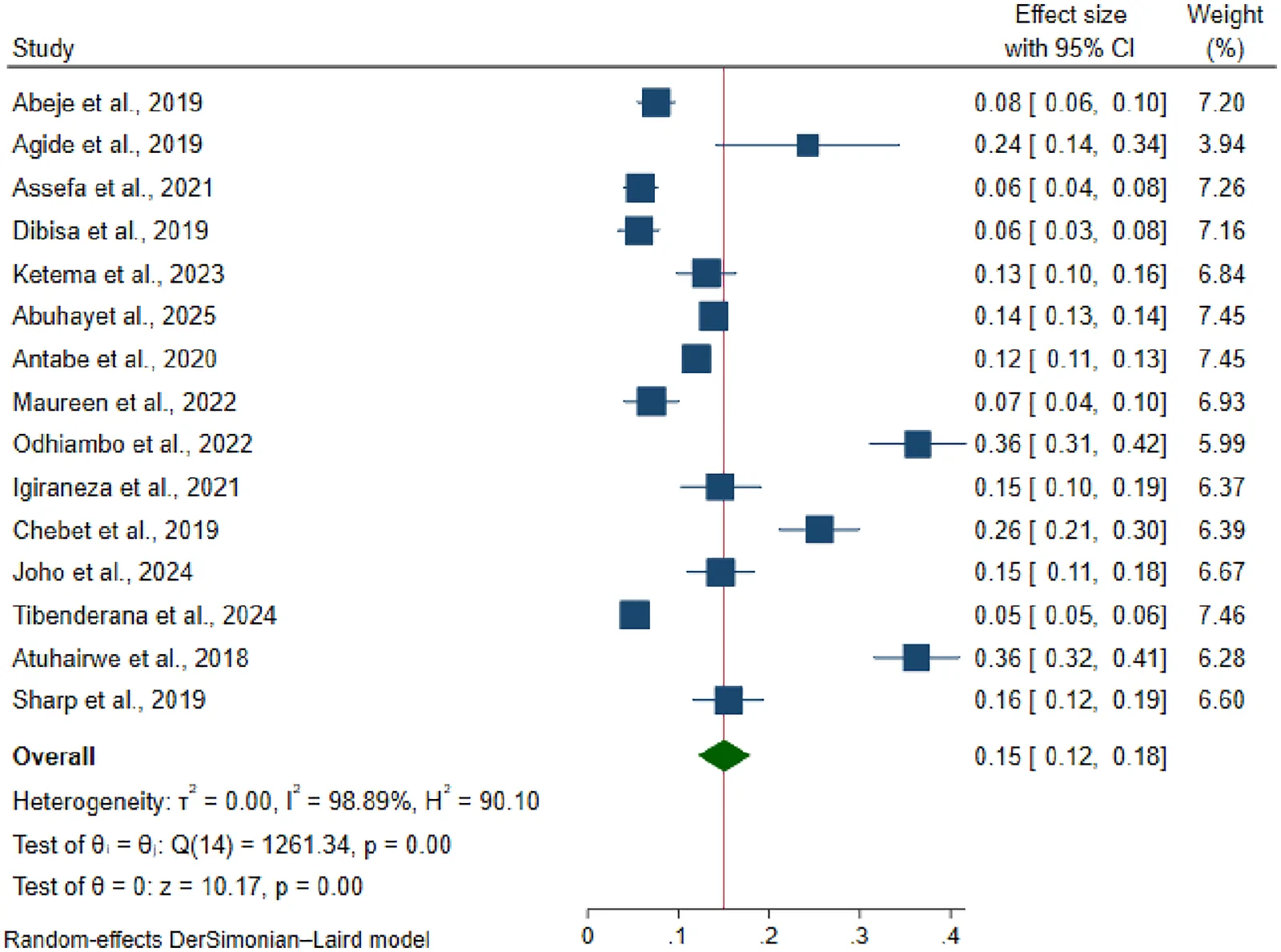
Forest plot for the pooled clinical breast examination uptake among women in East African countries, 2025.

### Publication bias

Publication bias was initially assessed through visual inspection of the funnel plot; the result indicated asymmetry, potentially indicating the presence of bias. Following that, Egger’s regression test was conducted, yielding a non-significant result (p-value = 0.985), thereby suggesting no statistically significant evidence of publication bias (S1 Fig).

### Managing heterogeneity

The pooled estimate of the random effect model indicated significant heterogeneity across included studies. As a result, to explore potential sources of heterogeneity, we conducted subgroup analysis and sensitivity analyses.

### Subgroup analyses

For this systematic review and meta-analysis, subgroup analyses were performed according to key study characteristics, including study country, study setting, place of residence, sample size, publication year, and risk of bias quality score (S2**–**S7 Fig).

The subgroup analysis revealed substantial difference of CBE uptake among countries. The pooled estimate of CBE uptake in Ethiopia was 9% ([95% CI:6%, 12%]; I^2^ = 85.7%, p-value < 0.001) whereas it was higher (26%) in Uganda (95% CI: 6%, 46%; I^2^ = 97.81%, p-value < 0.001).

Studies with lower sample size (< 500 participants) yielded a significant higher pooled uptake (19%) (95% CI: 13%, 26%; I^2^ = 96.6%, p-value < 0.001) than studies with higher sample size (>500 participants) (9%) (95% CI: 5%, 13%; I^2^ = 99.5%, p-value < 0.001).

Besides, a subgroup analysis was performed based on the year of publication. In studies published from 2015 to 2020, the pooled uptake of CBE was 16% (95% CI: 11%, 20%; I^2^ = 97.09%, p-value < 0.001). And, studies published between 2020 and 2025 showed a pooled uptake of CBE was 15% (95% CI: 10%, 19%; I^2^ = 99.3%, p-value < 0.001).

Furthermore, among studies rated as having a low risk of bias, the pooled CBE was 15% (95% CI: 11%, 18%; I^2^ = 99.1%, p-value < 0.001). In studies with a moderate risk of bias, the estimated uptake of CBE was 16% (95% CI: 13%, 20%; I^2^ = 33.8%, p-value < 0.221).

The subgroup analysis by place of residence was performed using two categories: urban and mixed (urban and rural) because none of studies reported findings from rural areas. Studies from mixed (urban and rural) residents had higher CBE uptake (17%) (95% CI: 10%, 24%; I^2^ = 99.82%, p-value < 0.001) than urban residents (13%) (95% CI: 6%, 20%; I^2^ = 96.08%, p-value < 0.001).

Studies conducted in community settings had higher pooled CBE uptake (16%) (95% CI: 9%, 23%; I^2^ = 99.80%, p-value < 0.001) compared to those conducted in institutional settings (15%) (95% CI: 8%, 22%; I^2^ = 94.98%, p-value < 0.001). However, high heterogeneity and insufficient included studies from few countries may reduce the statistical power of the result. Consequently, the overall pooled estimate should be interpreted with caution.

### Sensitivity analysis

Using a random-effects model, we assessed the influence of each study on the overall pooled estimate of clinical breast examination. The results indicated that no single study significantly affected the overall estimate, as the pooled estimated CBE remained stable when each study was sequentially omitted (S6 Fig).

### Factors with the uptake of clinical breast examination

A separate random-effects meta-analysis was conducted to assess the association between various covariates, such as age, residence, educational status, occupation, marital status, and awareness of breast cancer screening methods, and the uptake of CBE in East African countries. We calculated pooled effect sizes using adjusted odds ratios reported in the primary studies.

The analysis revealed that being aged 35–49 years, attaining higher education, and having awareness of breast cancer were significantly associated with increased CBE uptake, while rural residence was associated with lower uptake (Fig 3**).**

**Fig 3:**
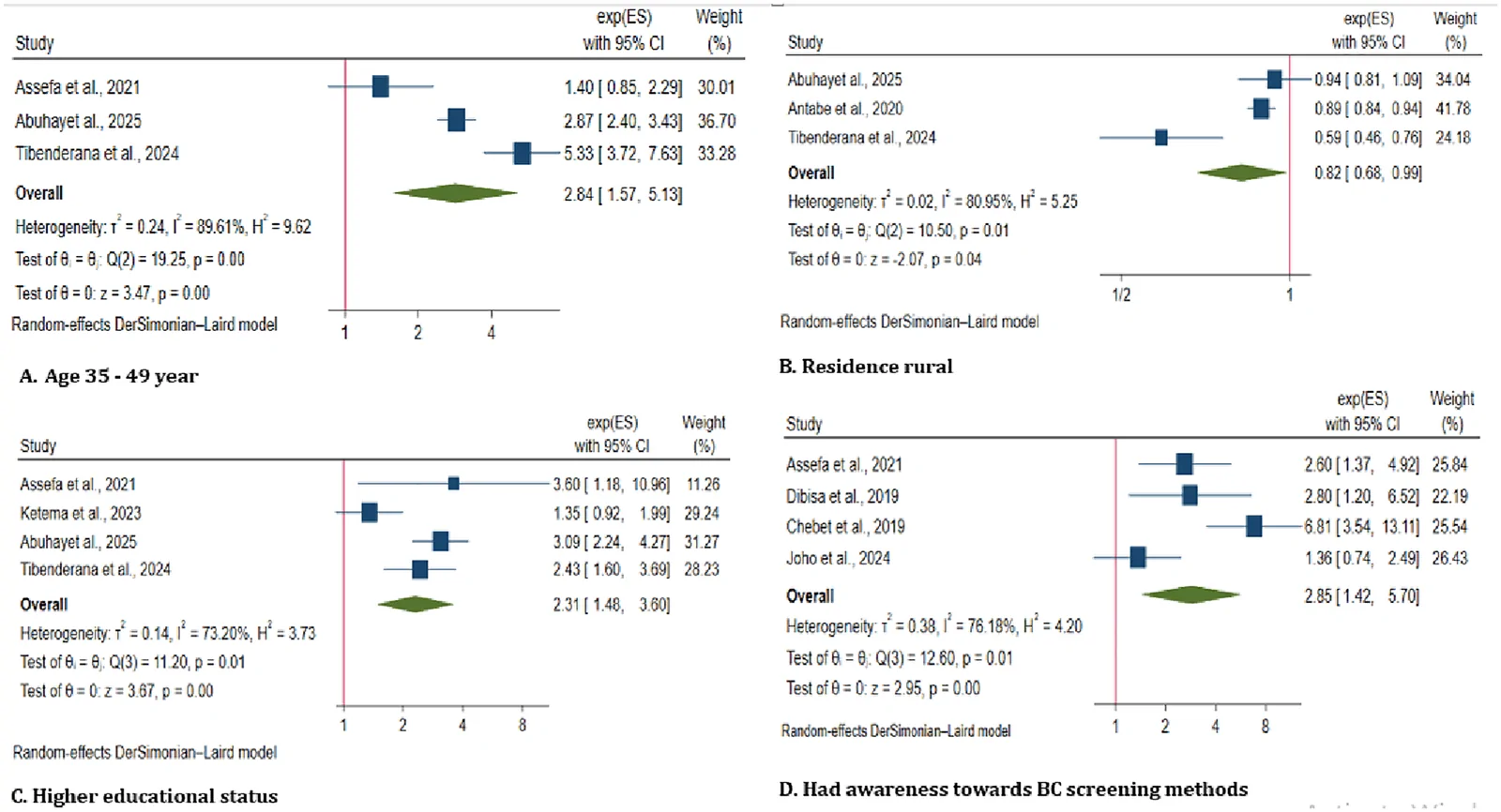
Factors associated with uptake of clinical breast examination among women in East African countries, 2025.

This study revealed that women aged 35–49 years had significantly higher odds of CBE uptake compared to those aged 15–34 years (AOR = 2.84; 95% CI: 1.57, 5.13). Women residing in rural areas had 18% less uptake of CBE compared to those living in urban areas (AOR = 0.82; 95% CI: 0.68, 0.99). Additionally, women with higher educational attainment had 2.31 times greater odds of CBE uptake compared to those with lower education levels (AOR = 2.31; 95% CI: 1.48, 3.60). Furthermore, women who were aware of breast cancer screening methods had significantly higher odds of CBE uptake than those who were not aware (AOR = 2.05; 95% CI: 1.23, 3.28) (**Table 2).**

**Table 2:** Pooled effect estimates of factors associated with clinical breast examination uptake in East African countries, 2025.

Variable	Category	AOR (95% CI)	Heterogeneity (I^2^, P-value)	Egger’s P-value	Total studies	Sample size
Age	15 - 34 years	1	Ref			
	35 - 49 years	2.84 (1.57, 5.13) *	(89.61%, 0.001)	0.930	3	32,472
Residence	Urban	1	Ref			
	Rural	0.82 (0.68, 0.99) *	(80.95%, 0.011)		3	46,579
Educational status	Lower educational status	1	Ref			
	Higher educational status	2.31 (1.48, 3.60) *	(73.2%, 0.011)	0.917	4	32,892
Occupation	Un-employed	1	Ref			
	Employed	1.30 (0.85, 2.01)	(54.51%, 0.091)	0.954	4	32,892
Marital status	Married	1	Ref			
	Single	1.00 (0.61, 1.64)	(72.32%, 0.011)	0.464	4	32,024
Awareness towards BC screening methods	No	1	Ref			
	Yes	2.05 (1.23, 3.28) *	(29.07%, 0.241)	0.761	3	1,794

* Statistically significant at 5% level, OR: Odds Ratio, CI: Confidence Interval, I^2^: I-squared.

## Discussion

Breast cancer remains the leading cause of cancer-related morbidity and mortality among women in East Africa. Although clinical breast examination (CBE) is a cost-effective and proven method for the early detection of breast cancer and reduction of associated mortality, its utilization across the region remains suboptimal. Furthermore, there is a paucity of comprehensive data on the prevalence of CBE uptake and the factors influencing its utilization. Therefore, this study aimed to assess the pooled uptake and associated factors of CBE in this region.

In this study, the pooled uptake of CBE among women in five countries in East Africa was 15% (95% CI: 12%, 18%) despite significant heterogeneity**.** This was lower than low- and middle-income countries (LMICs), where 23% of women had undergone breast cancer screening [39], and in Asian countries, where the prevalence was 24% [40]. The observed disparity can be attributed to differences in healthcare infrastructure, the availability and accessibility of screening services, levels of breast cancer awareness, cultural beliefs, and national health policies that prioritize cancer prevention and detection. Low coverage of CBE might be due to bottlenecks post screening continuum of care such as geographical distance from health institutions, high-out-of pocket diagnostic cost [41].

Possible reasons for low uptake in East Africa might be healthcare access barriers, financial constraints, difficulties in obtaining permission, and geographical challenges, such as long distances to health facilities [41,42].

This study also found considerable variation in the pooled uptake of CBE across countries in the subgroup analysis, ranging from 9% in Ethiopia to 26% in Uganda. These differences are likely attributable to variations in public awareness and cultural beliefs. For instance, in Ethiopia, only 56% of women demonstrated good knowledge about breast examination [43], whereas in Uganda, awareness levels were substantially higher, reaching 98% [44].

Moreover, this systematic review and meta-analysis identified several significant factors associated with the uptake of CBE in East African countries, including age, place of residence, educational status, and level of awareness about breast cancer.

The heterogeneity among individual studies remains high which might be due to methodological and contextual diversity. This high heterogeneity implies that the true uptake varies substantially across regions rather than reflecting a single pooled estimate. This variation might be occur due to the primary studies were different in geographical locations, sample sizes, study populations, policies, infrastructures and clinical settings. Consequently, overall pooled estimate should be interpreted with caution considering subgroup analysis report. The subgroup analysis identified key sources of variation including country of the study, sample size, study setting, publication year, place of residence and methodological quality score. This variability likely stems from cross-country differences in health care infrastructures, clinical protocols, and public health policies [45]. Furthermore, broad country level grouping might mask regional differences in the incorporated variables.

This study revealed that the uptake of CBE was higher among women with age 35–49 years compared to those 15–34 years old. This finding is supported by a study conducted in India, which revealed that the proportion of cancer screening increased as age increased with women’s age [46]. This could be because older women are more likely to perceive themselves as being at higher risk for breast cancer and are more engaged in CBE [47], both of which contribute to increased screening uptake.

Women residing in rural areas were less likely to undergo CBE compared to those living in urban areas. This result is consistent with a study conducted in eight selected countries, which revealed that rural women were less likely to participate in BC screening than their urban counterparts [48]. This could be related to the fact that rural women frequently face multiple barriers that negatively impact their access to health services. These include limited knowledge about available healthcare services [49], lower levels of health literacy [50], and geographic and economic barriers, which have a profound effect on healthcare access for women in rural areas [51]. Collectively, these factors may decrease the uptake of CBE services among rural dwellers.

Women with higher educational attainment had significantly higher CBE uptakes compared to those with lower levels of education. This finding is supported by a study conducted in Vietnam, which revealed that women with lower educational attainment were less likely to undergo CBE [52]. Similarly, evidence from the global level indicates a positive association between higher educational levels and utilization of breast cancer screening services [53]. This is because educated women are more likely to have better health literacy, which contributes to informed decision-making regarding preventive health behaviors.

Furthermore, in this study, women who were aware of breast cancer screening methods demonstrated higher uptakes of CBE compared to those who lacked such awareness. This finding is consistent with a study conducted in the United Arab Emirates [54], which reported that individuals who participated in breast cancer awareness programs were significantly more likely to utilize screening services. These findings highlight the importance of awareness creation in promoting early detection and preventative health habits among women as WHO recommended three pillars on breast cancer prevention including, health promotion for early detection, timely diagnosis and comprehensive breast cancer management [55].

This systematic review and meta-analysis have both strengths and limitations. Synthesizing regional data provides a comprehensive pooled estimate of CBE uptake, while incorporating low-risk of bias studies enhance internal validity and generalizability. The inclusion of studies with a low risk of bias strengthens internal validity and enhances the generalizability of the findings to similar settings. Notably, this study identified key associated factors of CBE uptake, contributing novel evidence to the existing body of literature and offering important insights toward achieving Sustainable Development Goal (SDG) 3.4 [17]. Limitations of this study include: incorporating only cross-sectional observational and pooling adjusted odds ratios from studies with different adjustment sets may limit the ability to infer causality resulting potential for residual confounding. Excluding qualitative research may also limit the contextual comprehensiveness of findings. As all included studies are cross-sectional designs, recall bias and social desirability bias may occurred. Furthermore, the generalizability of the pooled estimate is constrained as data was drawn from only five East Africa countries (Ethiopia, Kenya, Rwanda, Tanzania, and Uganda), and small number of included primary studies may reduce the statistical power of egger’s test. This review primarily reflects the context of Eastern and Horn-adjacent mainland Africa. There remains a critical evidence gap in the broader Horn of Africa and regional island nations, underscoring the urgent need for primary research in these underrepresented settings to inform comprehensive regional health policies.

## Conclusion

The pooled evidence suggests that the prevalence of clinical breast examination (CBE) in East Africa was low, highlighting a low uptake of this essential screening method across the region; however, these findings should be interpreted in light of notable limitations. Significant heterogeneity across the included studies, stemming from variations in study designs, as well as the mixing of large, nationally representative samples with smaller local cohorts creates a methodological dichotomy and data retrieved from only few countries may limit generalizability. Women aged 35–49 years who attained higher education, and women who had awareness of breast cancer screening methods, were more likely to undergo CBE. However, rural residence was negatively associated with the uptake of CBE. Integrated interventions across continuum of care are important, as measuring CBE uptake level alone is insufficient to reduce breast cancer morbidity and mortality. National policy makers and stakeholders should consider prioritizing raising awareness and embedding screening into primary healthcare, particularly for rural, younger, and lower educational level women. Consequently, such targeted public health strategies may contribute to achieving Sustainable Development Goal (SDG 3.4) by supporting the reduction of premature mortality from non-communicable diseases through early detection and linkage to treatment. Integrating community awareness campaigns on clinical breast examination (CBE) into packages delivered by community health workers, mobile outreach services, and nurse-led screening programs represents a viable approach.

## Supporting information

S1 Table Search Strategy in Included Databases, for uptake of Clinical breast examination among women in East African countries, 2025. (DOCX)

S2 Table Eligibility criteria for synthesis of CBE uptake and associated factors among women in East Africa using CoCoPop frame. (DOCX)

S3 Table Risk of bias assessment for cross-sectional studies. (DOCX)

S4 Table GRADE Evidence Profile for Clinical Breast Examination (CBE) Uptake and associated factors in East Africa. (DOCX)

S1 Fig Funnel plot showing publication bias for the pooled prevalence of clinical breast examination uptake in East African countries, 2025. (DOCX)

S2 Fig Subgroup analysis of clinical breast examination uptake by country among studies conducted in East African countries, 2025. (DOCX)

S3 Fig Subgroup analysis of clinical breast examination uptake by sample size of studies conducted in East African countries, 2025. (DOCX)

S4 Fig Subgroup analysis of clinical breast examination uptake by publication year of studies conducted in East African countries, 2025. (DOCX)

S5 Fig Subgroup analysis of clinical breast examination uptake by quality of primary studies conducted in East African countries, 2025. (DOCX)

S6 Fig Subgroup analysis of clinical breast examination uptake by place of residence conducted in East African countries, 2025. (DOCX)

S7 Fig Subgroup analysis of clinical breast examination uptake by study setting conducted in East African countries, 2025. (DOCX)

S8 Fig Leave-one-out sensitivity analysis assessing the influence of individual studies on the pooled estimate of clinical breast examination uptake in East African countries, 2025. (DOCX)

S1 Data Raw data spreadsheet containing minimal dataset including, variables, proportions, references categories, confidence intervals, and standard errors. (XLS)

S1 File PRISMA 2020 Checklist. (DOCX)

S1 Appendix Risk of Bias Assessment. Table A. Risk of bias assessment for cross-sectional studies. Table B. Risk of bias assessment for retrospective cohort study.
(DOCX)
