# Supplementary material for: Breast cancer examination uptake and its associated factors among women in East African countries: A systematic review and meta-analysis
Source: PLOS Glob Public Health. 2026 Nov 5;6(11):e0006727. doi: 10.1371/journal.pgph.0006727 (PMC13739099; doi:10.1371/journal.pgph.0006727)
Supplement: Supplementary file 1 [file pgph.0006727.s001.docx]

**S1 Table: Search Strategy in Included Databases, for uptake of Clinical breast examination among women in East Africa countries, 2025**

| **Data bases** | **Search strategy** |
| --- | --- |
| **PubMed** | ((("clinical breast examination"[All Fields]) OR ("clinical breast cancer screening"[All Fields]) OR "breast cancer screening" [All Fields] OR "Early Detection of Cancer" [MeSH Terms] OR "early detection of breast cancer"[All Fields] OR "breast neoplasms"[MeSH Terms]) AND (("Reproductive age women" [All Fields] OR "female"[All Fields]) OR ("women"[MeSH Terms] OR "women"[All Fields])) AND ((Burundi OR Comoros OR Djibouti OR Eritrea OR Ethiopia OR Kenya OR Madagascar OR Malawi OR Mauritius OR Mozambique OR Rwanda OR Seychelles OR Somalia OR "South Sudan" OR Tanzania OR Uganda OR Zambia OR Zimbabwe)))  Searched studies = **1,098** Filters applied: from 2015/1/1 - 2025/4/30. |
| Scopus | TITLE-ABS-KEY ("clinical breast examination") OR TITLE-ABS-KEY ("clinical breast cancer screening") OR TITLE-ABS-KEY ("breast cancer screening") OR TITLE-ABS-KEY ("Early Detection of Cancer") OR TITLE-ABS-KEY ("early detection of breast cancer") OR TITLE-ABS-KEY ("breast neoplasms" ) AND TITLE-ABS-KEY ("Reproductive age women") OR TITLE-ABS-KEY ( women ) AND TITLE-ABS-KEY (Burundi) OR TITLE-ABS-KEY (Comoros) OR TITLE-ABS-KEY (Djibouti) OR TITLE-ABS-KEY (Eritrea) OR TITLE-ABS-KEY (Ethiopia) OR TITLE-ABS-KEY (Kenya) OR TITLE-ABS-KEY (Madagascar) OR TITLE-ABS-KEY (Malawi) OR TITLE-ABS-KEY (Mauritius) OR TITLE-ABS-KEY ( Mozambique) OR TITLE-ABS-KEY (Rwanda) OR TITLE-ABS-KEY (Seychelles) OR TITLE-ABS-KEY ( Somalia) OR TITLE-ABS-KEY ("South Sudan" ) OR TITLE-ABS-KEY (Tanzania) OR TITLE-ABS-KEY ( Uganda) OR TITLE-ABS-KEY (Zambia) OR TITLE-ABS-KEY (Zimbabwe) AND PUBYEAR > 2014 AND PUBYEAR < 2026 AND ( LIMIT-TO ( SRCTYPE , "j" )) AND (LIMIT-TO (SUBJAREA , "MEDI") OR LIMIT-TO ( SUBJAREA , "NURS" ) OR LIMIT-TO ( SUBJAREA , "HEAL" )) AND (LIMIT-TO (DOCTYPE , "ar")) AND ( LIMIT-TO (LANGUAGE , "English" )) **=516** articles |
| Google scholar | **With all of word:** clinical breast cancer screening uptake and its associated factors in East Africa  **with the exact phrases:** clinical breast cancer screening OR clinical breast cancer examination  **At least one of those word**: Burundi OR Comoros OR Djibouti OR Eritrea OR Ethiopia OR Kenya OR Madagascar OR Malawi OR Mauritius OR Mozambique OR Rwanda OR Seychelles OR Somalia OR "South Sudan" OR Tanzania OR Uganda OR Zambia OR Zimbabwe  **where my words occur:** Anywhere in the article  **Return articles dated between**: 2015 – 2025  Searched article =303 articles |
| Research4life | Clinical breast cancer screening uptake and its associated factors among reproductive age women in East Africa countries  Content Type: Journal articles, Publication Date: 2015 – 2025, Discipline: Medicine and Public Health, Subject term: Breast cancer, Language: English. Searched articles: 56 |

Legend: AB: abstract, MH: MesH term, TI: title, TW: text word
