# Supplementary material for: Breast cancer examination uptake and its associated factors among women in East African countries: A systematic review and meta-analysis
Source: PLOS Glob Public Health. 2026 Nov 5;6(11):e0006727. doi: 10.1371/journal.pgph.0006727 (PMC13739099; doi:10.1371/journal.pgph.0006727)
Supplement: Supplementary file 2 [file pgph.0006727.s002.docx]

**S2 Table: The Joanna Briggs Institute (JBI) risk of bias assessment appraisal for cross-sectional studies.**

| **ID** | **Author** | **Publication Year** | **C1** | **C2** | **C3** | **C4** | **C5** | **C6** | **C7** | **C8** | **#Total score** | **Quality score** |
| --- | --- | --- | --- | --- | --- | --- | --- | --- | --- | --- | --- | --- |
| 1 | Abeje et al. | 2017 | Y | Y | Y | Y | U | U | Y | Y | 7 | Low risk |
| 2 | Agide et al. | 2020 | Y | N | U | Y | U | U | Y | Y | 5.5 | Moderate risk |
| 3 | Assefa et al. | 2022 | Y | Y | U | Y | U | U | Y | Y | 6.5 | Low risk |
| 4 | Dibisa et al. | 2023 | Y | Y | Y | N | U | U | Y | Y | 6 | Low risk |
| 5 | Ketema et al. | 2022 | Y | Y | Y | Y | U | U | Y | Y | 7 | Low risk |
| 6 | Abuhay et al. | 2021 | Y | N | Y | Y | U | U | Y | Y | 6 | Low risk |
| 7 | Antabe et al. | 2019 | Y | Y | Y | Y | U | U | Y | Y | 7 | Low risk |
| 8 | Maureen et al. | 2021 | Y | Y | U | Y | U | U | Y | Y | 6.5 | Low risk |
| 9 | Odhiambo et al. | 2021 | Y | Y | Y | Y | U | U | Y | Y | 7 | Low risk |
| 10 | Igiraneza et al. | 2024 | Y | Y | N | Y | U | U | N | Y | 5 | Moderate risk |
| 11 | Chebet et al. | 2022 | Y | Y | Y | Y | U | U | Y | Y | 7 | Low risk |
| 12 | Joho et al. | 2023 | Y | Y | Y | N | U | U | Y | Y | 6 | Moderate risk |
| 13 | Tibenderana et al. | 2018 | Y | Y | U | Y | U | U | Y | Y | 6.5 | Low risk |
| 14 | Atuhairwe et al. | 2015 | Y | Y | Y | Y | U | U | Y | Y | 7 | Low risk |
| 15 | Sharp et al. | 2021 | Y | Y | N | Y | U | N | Y | Y | 5.5 | Moderate risk |

Y = Yes; N = No; U – Unclear; N/A - Not/Applicable

C1: Were the criteria for inclusion in the sample clearly defined? C2: Were the study subjects and the setting described in detail? C3: Was the exposure measured in a valid and reliable way? C4: Were objective, standard criteria used for measurement of the condition? C5: Were confounding factors identified? C6: Were strategies to deal with confounding factors stated?

C7: Were the outcomes measured in a valid and reliable way? C8: Was appropriate statistical analysis used?

Summarizing Scores

“Yes” was given a value of 1, the score “No” was given a value of 0, the score “Unclear” was given a value of 0.5, and “Not applicable was denoted as N/A

Interpreting the Scores

Low risk of bias (High quality) was nominated if the study scored 6 and above, moderate risk of bias (Good quality) if the study scored between 5 and 4, and High risk of bias (lower quality) for studies scored below 4.
