# Supplementary material for: Breast cancer examination uptake and its associated factors among women in East African countries: A systematic review and meta-analysis
Source: PLOS Glob Public Health. 2026 Nov 5;6(11):e0006727. doi: 10.1371/journal.pgph.0006727 (PMC13739099; doi:10.1371/journal.pgph.0006727)
Supplement: Supplementary file 3 [file pgph.0006727.s003.docx]

S3 Table: Eligibility criteria for synthesis of CBE uptake and associated factors among women in East Africa using CoCoPop framework

| **Domain/ Criterion** | **Inclusion Criteria** | **Exclusion Criteria** |
| --- | --- | --- |
| **Population (Condition & Context)** | Women residing in East African countries | Studies conducted outside of Africa |
|  | Specific target demographic women of reproductive age 15–49 women | hospitalized patients with severe co-morbidities |
| **Study Design** | Observational study designs | Experimental studies (RCTs, quasi-experimental), qualitative studies, case reports/series, systematic reviews, editorials, commentaries, qualitative studies and conference abstracts without full text. |
| **Outcomes of Interest** | Primary studies reporting CBE uptake prevalence | Studies lacking sufficient numeric data to extract or recalculate overall prevalence or measure of association |
|  | Studies reporting Adjusted odds ratios [AOR] for associated factors |  |
| **Publication & Language** | Published peer-reviewed articles and eligible gray literature | Articles without available full text after reasonable author contact attempts. |
|  | Articles published in any language | Duplicate publications, anonymous reports, or overlapping datasets. |
| **Quality / Risk of Bias** | Studies meeting low or moderate risk of bias | Studies categorized as high risk of bias or low methodological quality upon critical appraisal. |
