# Supplementary material for: Breast cancer examination uptake and its associated factors among women in East African countries: A systematic review and meta-analysis
Source: PLOS Glob Public Health. 2026 Nov 5;6(11):e0006727. doi: 10.1371/journal.pgph.0006727 (PMC13739099; doi:10.1371/journal.pgph.0006727)
Supplement: Supplementary file 4 [file pgph.0006727.s004.docx]

**S4 Table:** GRADE Evidence Profile for Clinical Breast Examination (CBE) Uptake and Determinants in East Africa

| **Sr. No** | **Outcome / Factors** | **No. of Studies (Participants)** | **Prevalence (95% CI)** | **Certainty Assessment**  **(Risk of Bias, Inconsistency, Indirectness, Imprecision, Publication Bias)** | **Certainty of Evidence (GRADE)** |
| --- | --- | --- | --- | --- | --- |
| 1 | Pooled Uptake of CBE | 15 studies (35,911 participants) | Prevalence: 15% (12%, 18%) | Serious risk of bias (cross-sectional design); Very serious inconsistency (I^2^ = 98.89%);  No indirectness; Not serious imprecision;  No publication bias detected (Egger's p = 0.985) | Very Low |
| 2 | Age (35–49 versus 15–34 years) | 3 studies (32,472 participants) | OR = 2.84 (1.57, 5.13) | Serious risk of bias; Serious inconsistency (I^2^ = 89.61%); No indirectness; Not serious imprecision; Egger's p = 0.930 | Low |
| 3 | Residence (Rural versus Urban) | 3 studies (46,579 participants) | OR = 0.82 (0.68, 0.99) | Serious risk of bias; Serious inconsistency (I^2^ = 80.95%); No indirectness; Not serious imprecision. | Low |
| 4 | Educational Status (Higher versus Lower) | 4 studies (32,892 participants) | OR = 2.31 (1.48, 3.60) | Serious risk of bias; Serious inconsistency (I^2^ = 73.2%); No indirectness; Not serious imprecision; Egger's p = 0.917 | Low |
| 5 | Awareness of Screening Methods  (Yes versus No) | 3 studies (1,794 participants) | OR = 2.05 (1.28, 3.28) | Serious risk of bias; Low inconsistency (I^2^ = 29.07%); No indirectness;  Not serious imprecision;  Egger's p = 0.761. | Low |
