# Supplementary material for: Breast cancer examination uptake and its associated factors among women in East African countries: A systematic review and meta-analysis
Source: PLOS Glob Public Health. 2026 Nov 5;6(11):e0006727. doi: 10.1371/journal.pgph.0006727 (PMC13739099; doi:10.1371/journal.pgph.0006727)
Supplement: Supplementary file 6 [file pgph.0006727.s006.docx]

**S2 Fig: Subgroup analysis of clinical breast examination uptake by country among studies conducted in East African countries, 2025.**
