# Supplementary material for: Breast cancer examination uptake and its associated factors among women in East African countries: A systematic review and meta-analysis
Source: PLOS Glob Public Health. 2026 Nov 5;6(11):e0006727. doi: 10.1371/journal.pgph.0006727 (PMC13739099; doi:10.1371/journal.pgph.0006727)
Supplement: Supplementary file 14 [file pgph.0006727.s015.docx]

**S1 Appendix. Risk of Bias Assessment**

**Table A: Risk of bias assessment for cross-sectional studies.**

| **ID** | **Author** | **Year** | **C1** | **C2** | **C3** | **C4** | **C5** | **C6** | **C7** | **C8** | **#Total score** | **Quality score** |
| --- | --- | --- | --- | --- | --- | --- | --- | --- | --- | --- | --- | --- |
| 1 | Chisha et al. | 2017 | Y | Y | Y | U | N/A | N/A | Y | Y | 5.5 | Low risk |
| 2 | Tilahun et al. | 2020 | Y | N | Y | Y | N/A | N/A | Y | Y | 6 | Low risk |
| 3 | Alemu Mersha et al. | 2022 | Y | Y | U | Y | N/A | N/A | Y | Y | 5.5 | Low risk |
| 4 | Zegeye et al. | 2023 | Y | Y | Y | N | N/A | N/A | Y | Y | 5 | Low risk |
| 5 | Sahiledengle et al. | 2022 | Y | Y | Y | Y | N/A | N/A | Y | Y | 6 | Low risk |
| 6 | Seid et al. | 2021 | Y | Y | Y | Y | N/A | N/A | Y | Y | 6 | Low risk |
| 7 | Shibru et al. | 2019 | Y | Y | Y | Y | N/A | N/A | Y | Y | 6 | Low risk |
| 8 | Ejigu and Tsegaw | 2021 | Y | N | Y | Y | N/A | N/A | Y | Y | 5 | Low risk |
| 9 | Tsegaw et al. | 2021 | Y | Y | U | Y | N/A | N/A | Y | Y | 5.5 | Low risk |
| 10 | Ireri et al. | 2024 | Y | Y | Y | Y | N/A | N/A | Y | Y | 6 | Low risk |
| 11 | Musawa et al. | 2022 | Y | Y | Y | Y | N/A | N/A | Y | Y | 6 | Low risk |
| 12 | Nyakaba et al. | 2023 | U | Y | Y | N | N/A | N/A | Y | Y | 4.5 | Moderate risk |
| 13 | MoH Kenya | 2018 | Y | Y | Y | Y | N/A | N/A | Y | Y | 6 | Low risk |
| 14 | Burgess et al. | 2015 | Y | Y | Y | Y | N/A | N/A | Y | Y | 6 | Low risk |
| 15 | Niyodusenga A et al. | 2021 | Y | Y | Y | Y | N/A | N/A | Y | Y | 6 | Low risk |
| 16 | Iradukunda et al. | 2021 | Y | Y | Y | Y | N/A | N/A | Y | Y | 6 | Low risk |
| 17 | Sube et al. | 2020 | Y | Y | Y | U | N/A | N/A | Y | Y | 5.5 | Low risk |
| 18 | Cleland et al. | 2016 | Y | Y | Y | Y | N/A | N/A | Y | Y | 6 | Low risk |
| 19 | Magan et al. | 2019 | Y | N | Y | Y | N/A | N/A | Y | Y | 5 | Low risk |
| 20 | Seba et al. | 2015 | Y | Y | Y | Y | N/A | N/A | Y | Y | 6 | Low risk |
| 21 | Lewis et al. | 2018 | Y | Y | Y | Y | N/A | N/A | Y | Y | 6 | Low risk |

Y = Yes; N = No; U – Unclear; N/A - Not/Applicable

C1: Were the criteria for inclusion in the sample clearly defined? C2: Were the study subjects and the setting described in detail? C3: Was the exposure measured in a valid and reliable way? C4: Were objective, standard criteria used for measurement of the condition? C5: Were confounding factors identified? C6: Were strategies to deal with confounding factors stated?

C7: Were the outcomes measured in a valid and reliable way? C8: Was appropriate statistical analysis used?

Summarizing Scores

“Yes” was given a value of 1, the score “No” was given a value of 0, the score “Unclear” was given a value of 0.5, and “Not applicable was denoted as N/A

Interpreting the Scores

Low risk of bias (High quality) was nominated if the study scored 6 and above, moderate risk of bias (Good quality) if the study scored between 5 and 4, and High risk of bias (lower quality) for studies scored below 3.

**Table B: Risk of bias assessment for retrospective cohort study**

| **ID** | **Author** | **Year** | **C1** | **C2** | **C3** | **C4** | **C5** | **C6** | **C7** | **C8** | **C9** | **C10** | **C11** | **#Total score** | **Quality score** |
| --- | --- | --- | --- | --- | --- | --- | --- | --- | --- | --- | --- | --- | --- | --- | --- |
| 1 | Debele et al. | 2021 | Y | Y | Y | N/A | N/A | Y | Y | Y | Y | Y | Y | 9 | Low risk |
| 2 | Gelcho and Gari | 2022 | Y | U | Y | N/A | N/A | Y | Y | Y | Y | U | Y | 8 | Low risk |
| 3 | Takele et al. | 2022 | Y | U | Y | N/A | N/A | Y | Y | Y | Y | Y | Y | 9 | Low risk |
| 4 | Azeze et al. | 2018 | Y | Y | Y | N/A | N/A | Y | Y | Y | Y | Y | Y | 9 | Low risk |
| 5 | Tassew et al. | 2023 | Y | U | U | N/A | N/A | Y | Y | Y | Y | N | Y | 8 | Low risk |
| 6 | Olwendo et al. | 2020 | Y | Y | Y | N/A | N/A | Y | Y | Y | Y | Y | Y | 9 | Low risk |
| 7 | Rigato et al. | 2022 | Y | Y | U | N/A | N/A | Y | Y | Y | Y | Y | Y | 8 | Low risk |
| 8 | Lewis et al. | 2022 | Y | U | Y | N/A | N/A | Y | Y | Y | Y | Y | Y | 8 | Low risk |

Y = Yes; N = No; U – Unclear; N/A - Not/Applicable

C1: Were the two groups similar and recruited from the same population? C2: Were the exposures measured similarly to assign people to both exposed and unexposed groups? C3: Was the exposure measured in a valid and reliable way? C4: Were confounding factors identified? C5: Were strategies to deal with confounding factors stated? C6: Were the groups/participants free of the outcome at the start of the study (or at the moment of exposure)? C7: Were the outcomes measured in a valid and reliable way? C8: Was the follow up time reported and sufficient to be long enough for outcomes to occur? C9: Was follow up complete, and if not, were the reasons to loss to follow up described and explored? C10: Were strategies to address incomplete follow up utilized? C11: Was appropriate statistical analysis used?

Summarizing Scores

“Yes” was given a value of 1, the score “No” was given a value of 0, the score “Unclear” was given a value of 0.5, and “Not applicable was denoted as N/A

Interpreting the Scores

Low risk of bias (High quality) was nominated if the study scored 8 and above, moderate risk of bias (Good quality) if the study scored between 7 and 5.5, and High risk of bias (lower quality) for studies scored below 5.
